# Vasoactive breathing manoeuvres as an effective coronary vasodilator in patients with suspected coronary artery disease: a prospective comparative oxygenation-sensitive CMR study

**DOI:** 10.1093/ehjimp/qyaf141

**Published:** 2025-11-13

**Authors:** Simran Shergill, Elizabeth Hillier, Anju Velvet, Charley A Budgeon, Aida Moafi, Mohamed Elshibly, Rachel England, Kelly S Parke, Joanne V Wormleighton, David Adlam, Sandeep S Hothi, Andrew Ladwiniec, Matthias G Friedrich, Gerry P McCann, J Ranjit Arnold

**Affiliations:** Department of Cardiovascular Sciences, University of Leicester, the National Institute for Health and Care Research Leicester Biomedical Research Centre and British Heart Foundation Centre of Research Excellence, Glenfield Hospital, Groby Road, Leicester LE3 9QP, UK; Department of Medicine, McGill University Health Centre, 3605 de la Montagne, Montreal, QC H3G 2M1, Canada; Department of Cardiovascular Sciences, University of Leicester, the National Institute for Health and Care Research Leicester Biomedical Research Centre and British Heart Foundation Centre of Research Excellence, Glenfield Hospital, Groby Road, Leicester LE3 9QP, UK; Cardiovascular Epidemiology Research Centre, School of Population and Global Health, University of Western Australia, 35 Stirling Highway, Crawley 6009, Australia; Department of Cardiovascular Sciences, University of Leicester, the National Institute for Health and Care Research Leicester Biomedical Research Centre and British Heart Foundation Centre of Research Excellence, Glenfield Hospital, Groby Road, Leicester LE3 9QP, UK; Department of Cardiovascular Sciences, University of Leicester, the National Institute for Health and Care Research Leicester Biomedical Research Centre and British Heart Foundation Centre of Research Excellence, Glenfield Hospital, Groby Road, Leicester LE3 9QP, UK; Department of Cardiovascular Sciences, University of Leicester, the National Institute for Health and Care Research Leicester Biomedical Research Centre and British Heart Foundation Centre of Research Excellence, Glenfield Hospital, Groby Road, Leicester LE3 9QP, UK; Department of Cardiovascular Sciences, University of Leicester, the National Institute for Health and Care Research Leicester Biomedical Research Centre and British Heart Foundation Centre of Research Excellence, Glenfield Hospital, Groby Road, Leicester LE3 9QP, UK; Department of Cardiovascular Sciences, University of Leicester, the National Institute for Health and Care Research Leicester Biomedical Research Centre and British Heart Foundation Centre of Research Excellence, Glenfield Hospital, Groby Road, Leicester LE3 9QP, UK; Department of Cardiovascular Sciences, University of Leicester, the National Institute for Health and Care Research Leicester Biomedical Research Centre and British Heart Foundation Centre of Research Excellence, Glenfield Hospital, Groby Road, Leicester LE3 9QP, UK; Heart and Lung Centre, Royal Wolverhampton NHS Trust, Wolverhampton Road, Wolverhampton WV10 0QP, UK; Institute of Cardiovascular Sciences, College of Medical and Dental Sciences, University of Birmingham, Edgbaston, Birmingham B15 2TT, UK; Department of Cardiovascular Sciences, University of Leicester, the National Institute for Health and Care Research Leicester Biomedical Research Centre and British Heart Foundation Centre of Research Excellence, Glenfield Hospital, Groby Road, Leicester LE3 9QP, UK; Department of Cardiology, University Hospitals of Leicester NHS Trust, Glenfield Hospital, Groby Road, Leicester LE3 9QP, UK; Department of Medicine, McGill University Health Centre, 3605 de la Montagne, Montreal, QC H3G 2M1, Canada; Department of Cardiovascular Sciences, University of Leicester, the National Institute for Health and Care Research Leicester Biomedical Research Centre and British Heart Foundation Centre of Research Excellence, Glenfield Hospital, Groby Road, Leicester LE3 9QP, UK; Department of Cardiovascular Sciences, University of Leicester, the National Institute for Health and Care Research Leicester Biomedical Research Centre and British Heart Foundation Centre of Research Excellence, Glenfield Hospital, Groby Road, Leicester LE3 9QP, UK

**Keywords:** oxygenation-sensitive, myocardial oxygenation, hyperventilation breath-hold, blood oxygen level-dependent, stable angina, fractional flow reserve

## Abstract

**Aims:**

In the assessment of myocardial ischaemia, vasoactive breathing manoeuvres have been proposed as a potential alternative to pharmacological vasodilator stress. However, it remains unclear whether the resultant physiological responses are comparable to those induced by adenosine in patients with suspected coronary artery disease (CAD). We sought to compare the myocardial oxygenation responses to a hyperventilation breath-hold (HVBH) manoeuvre with those elicited by adenosine, using oxygenation-sensitive cardiovascular magnetic resonance (OS-CMR).

**Methods and results:**

Patients with suspected angina underwent 3-Tesla OS-CMR with (i) HVBH and (ii) adenosine (140–210 μg/kg/min) prior to invasive coronary angiography. The primary outcome was the maximal percentage change in OS-CMR signal intensity for (i) HVBH [breathing-induced myocardial oxygenation reserve (B-MORE_max_)] and (ii) adenosine [adenosine-induced myocardial oxygenation reserve (A-MORE)]. Significant CAD was defined as fractional flow reserve ≤0.80 in epicardial vessels ≥2 mm diameter. From 53 prospectively recruited patients, 44 had complete, paired OS-CMR sequences (mean age 69 ± 9 years, 75% male, CAD prevalence 59%). The HVBH manoeuvre elicited a myocardial oxygenation response comparable to that of adenosine (B-MORE_max_ 7.0 ± 9.5% vs. A-MORE 8.6 ± 12.3%, mean difference: −1.6% [−6.0%, 2.8%]; *P* = 0.469), which remained similar at the segmental level following adjustment for age, sex, cardiovascular comorbidities, CAD, and infarction (mean difference: −1.7% [−4.0%, 0.5%]; *P* = 0.126).

**Conclusion:**

In patients with suspected CAD, HVBH induces a myocardial oxygenation response comparable to that elicited by adenosine. Future prospective studies evaluating the diagnostic accuracy of OS-CMR combined with vasoactive breathing manoeuvres for detecting significant CAD are warranted.

## Graphical Abstract

**Graphical Abstract:**
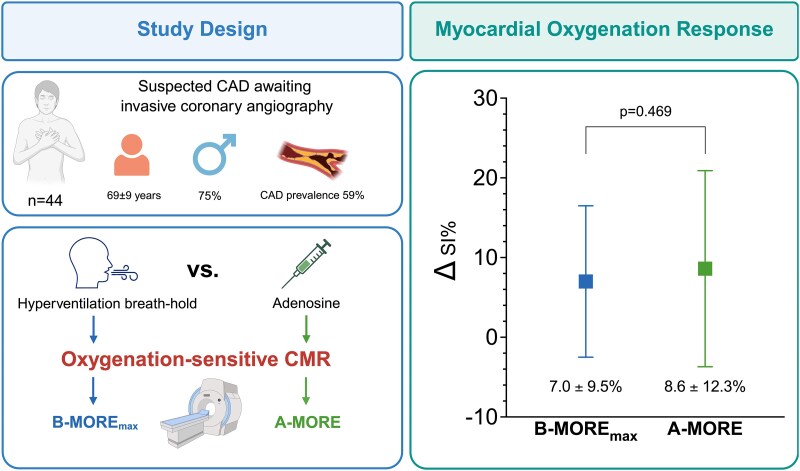
Data presented as mean ± standard deviation. Abbreviations: A-MORE, adenosine-induced myocardial oxygenation reserve; B-MORE_max_, breathing-induced myocardial oxygenation reserve (defined by the maximal signal intensity change during the post-hyperventilation breath-hold); CAD, coronary artery disease; CMR, cardiovascular magnetic resonance; SI, signal intensity.

## Introduction

In the assessment of patients with suspected coronary artery disease (CAD), the role of stress perfusion cardiovascular magnetic resonance (CMR) is well established.^1–5^ First-pass perfusion provides excellent diagnostic accuracy for the detection of myocardial ischaemia.^6^ However, its reliance on intravenous vasodilators and gadolinium-based contrast agents increases cost, carries a small yet significant risk of adverse effects, necessitating medical supervision during administration, and is contraindicated in certain patient groups.^7,8^ Hence, there are clear incentives to develop alternative, ‘needle-free’ approaches for accurate ischaemia detection.

Oxygenation-sensitive (OS) CMR exploits the inherent magnetic properties of haemoglobin: the transition of diamagnetic oxyhaemoglobin to paramagnetic deoxyhaemoglobin induces magnetic field inhomogeneities, resulting in a loss of signal intensity (SI) on T2*-weighted images, providing an endogenous source of contrast.^9–11^ Adenosine OS-CMR has been validated against invasive coronary angiography (ICA) for the detection of obstructive CAD, with favourable diagnostic performance.^12–16^ Vasoactive breathing manoeuvres have emerged as a non-pharmacological alternative for stress testing, capitalizing on the potent vasodilatory effects of carbon dioxide to induce a ‘luxury perfusion’ state similar to that induced by adenosine.^17,18^ Clinical studies have demonstrated that a protocol combining paced hyperventilation (HV) followed by a maximal voluntary breath-hold induces significant myocardial oxygenation changes detectable by OS-CMR,^19–21^ with feasibility demonstrated across diverse patient populations.^22–26^

Despite the potential of this drug-free, contrast-free protocol, it remains unclear whether the hyperventilation breath-hold (HVBH) manoeuvre elicits a myocardial oxygenation response comparable to that induced by adenosine in a patient population with a high prevalence of cardiovascular comorbidities, prescription medication use, and CAD. In this prospective clinical study, we sought to compare the myocardial oxygenation response of the HVBH manoeuvre with that of adenosine in patients with suspected CAD.

## Methods

### Study design

Fifty-three consecutive adult patients with *de novo* suspected angina clinically referred for diagnostic ICA were prospectively recruited from a single tertiary cardiac centre (Glenfield Hospital, Leicester, UK) between December 2023 and January 2025. Exclusion criteria were recent myocardial infarction (≤6 months), unstable angina, previous revascularisation, contraindications to adenosine (second/third-degree atrioventricular block, severe chronic obstructive pulmonary disease, moderate-severe asthma), severe renal dysfunction (estimated glomerular filtration rate <30 mL/min/1.73 m^2^), severe claustrophobia, and absolute contraindications to CMR (non-conditional cardiac implantable electronic device, pregnancy, ferromagnetic implants/foreign bodies). Participants were enrolled from the CONCORD study and invited to participate in this additional substudy (NCT04761991).^27^ The study was approved by the UK National Research Ethics Service (REC reference 19/EM/0295) and conducted in accordance with the Declaration of Helsinki, with all participants providing written informed consent prior to participation.

### Cardiovascular magnetic resonance

Prior to ICA, patients underwent research CMR at 3-Tesla (Vida or Skyra, Siemens Healthineers, Erlangen, Germany) with electrocardiographic (ECG) gating and an 18-channel phased-array cardiac receiver coil. Participants were advised to abstain from caffeine-containing products for at least 12 h prior to vasodilator stress, but routine anti-anginal medications (e.g. beta blockers and oral nitrates) were continued.

Research CMR consisted of:

1. Functional cine imaging in the three long-axis views (4, 2, 3-chamber) and a contiguous short-axis stack covering the ventricles from base to apex utilising a breath-hold balanced steady-state free precession pulse sequence (typical sequence parameters: echo time (TE) 1.49 ms, repetition time (TR) 47.5 ms, slice thickness 8 mm, distance factor 25%, matrix 256 × 166, field of view (FOV) 360–400 mm, FOV phase 81.3%, flip angle 80°). In those with poor breath-holding, a real-time, retrogated, steady-state free precession cine sequence was utilised.^28^
2. OS-CMR was acquired using a modified balanced steady-state free precession pulse sequence in a single, matched basal-mid ventricular slice (typical sequence parameters: TE 1.73 ms, TR 3.45 ms, slice thickness 10 mm, voxel size 2 × 2 × 10 mm^3^, matrix 192 × 120, bandwidth 1302 Hz/pixel, FOV 360–400 mm, FOV phase 75%, flip angle 35°), with shimming centred and tightly planned around the left ventricle to minimise artefact.^29^
  - HVBH OS-CMR: at baseline, an end-expiratory breath-hold OS-CMR sequence was acquired. Each participant then underwent a coached period of paced HV (30–35 breaths/min) for 60 s followed by a maximal voluntary end-expiratory breath-hold whilst a continuous OS-CMR sequence was acquired. A rest period of at least 3 min followed resumption of normal breathing to allow the participant to return to baseline haemodynamics.
  - Adenosine OS-CMR: a baseline, end-expiratory breath-hold OS-CMR sequence was then repeated prior to administering vasodilator stress. Adenosine was then infused at a rate of 140 μg/kg/min for 3–5 min. Subjects were monitored for symptoms throughout the infusion, with dose escalations at 2-min intervals to 170–210 μg/kg/min if there was an insufficient symptomatic and/or haemodynamic response (heart rate increase ≥10 beats per minute).^30^ At peak stress, a breath-hold OS-CMR acquisition was repeated.
3. Contrast-enhanced scar imaging utilized a breath-hold, T1-weighted segmented inversion-recovery sequence acquired 5–10 min after injection of a total of 0.15 mmol/kg gadoterate meglumine (Dotarem, Guerbet, France or Clariscan, GE HealthCare, IL, USA), with matched slice prescriptions to the long- and short-axis cine imaging (typical sequence parameters: TE 1.89 ms, slice thickness 8 mm, distance factor 25%, matrix 256 × 152, FOV 360–420 mm, FOV phase 80.5%, flip angle 20°). In tiring participants or those struggling with breath-holds, a free-breathing, single-shot inversion-recovery gradient echo sequence was used (typical sequence parameters: TE 1.17 ms, slice thickness 8 mm, distance factor 25%, matrix 224 × 148, FOV 340–380 mm, FOV phase 81.3%, flip angle 40°). A Look-Locker sequence was used to determine the optimal inversion time from the nulling of non-infarcted myocardium.

### Image analysis

CMR images were analysed offline, blinded to participant and angiographic details by a single observer (>3 years’ experience), using certified software (cvi42, v. 6.1.2; Circle Cardiovascular Imaging, Calgary, Canada). Volumetric analysis was performed with methods as previously described.^27^ OS-CMR and late gadolinium enhancement (LGE) images were analysed using the 16-segment American Heart Association model, with myocardial segments ascribed a coronary territory according to standard criteria.^31^ For qualitative LGE assessment, scar patterns were graded at a segmental level: 0 = normal, 1 = subendocardial, 2 = transmural, 3 = non-ischaemic, 4 = insertion point fibrosis. For quantitative assessment of infarction, the full-width at half-maximum technique was used.^32^

### OS-CMR analysis

For OS-CMR, the end-systolic (ES) phase was chosen for analysis as previously described.^29^ Image quality was graded on a 4-point scale: 3 = excellent, 2 = good, 1 = moderate, and 0 = unanalysable.^20^ Left ventricular endocardial and epicardial contours were manually drawn for each ES phase during each breath-hold acquisition, with a 10% offset applied to each border to minimise the risk of blood pool inclusion or partial volume effects. Mean myocardial segmental SI was calculated and recorded for: (i) baseline sequences prior to HVBH and adenosine administration, (ii) each ES phase during the maximal voluntary breath-hold following HV, and (iii) ES phase of peak adenosine stress. Segments with severe artefact (e.g. motion, off-resonance or susceptibility artefacts) were excluded from analysis.

The maximal percentage change in SI (ΔSI%) to adenosine [adenosine-induced myocardial oxygenation reserve (A-MORE)], HV [hyperventilation-induced myocardial oxygenation reserve (HV-MORE)], and HVBH [breathing-induced myocardial oxygenation reserve (B-MORE_max_)] was calculated as follows:


$$A-MORE\;=\;\left(\frac{Peak\;adenosine\;SI-Baseline\;adenosine\;SI}{Baseline\;adenosine\;SI}\right)\times \;100$$

$$HV-MORE\;=\;\left(\frac{Post\;HV\;SI-Pre\;HV\;SI}{Pre\;HV\;SI}\right)\times \;100$$

$$B-MORE_{max}\;=\;\left(\frac{Peak\;breath\;hold\;SI-Post\;HV\;SI}{Post\;HV\;SI}\right)\times \;100$$

Due to potential coronary flow heterogeneities in CAD, the B-MORE response may not be homogeneous or maximal at a prespecified time point (e.g. 30 s). Therefore, the ES phase with the maximal global ΔSI% during the breath-hold (B-MORE_max_) was used for the primary analysis, along with the corresponding territory and segmental mean SI values during that phase.

### ICA protocol and analysis

Clinically indicated ICA was performed according to standard clinical protocols by interventionalists blinded to imaging findings. Fractional flow reserve (FFR) was performed in epicardial vessels with visually determined stenosis of 40–90% using an intracoronary pressure wire (PressureWire X, Abbott Vascular, IL, USA) and 6-French guide catheter. Hyperaemia was induced with intravenous adenosine, and FFR was calculated as the ratio of mean distal coronary pressure to mean aortic pressure, adjusted for pressure drift.^33^ Significant CAD was defined as FFR ≤ 0.80 in an epicardial vessel ≥2 mm diameter. In vessels deemed not safe to perform FFR (subtotal or complete occlusions), the vessel was assumed to have an FFR of 0.50. In remaining vessels in which FFR had not been determined and with visually ≥25% (mild) stenosis, quantitative flow ratio (QFR) computation was performed offline by an independent observer, blinded to imaging and clinical details, using QFR v2.2 software (Medis Medical Imaging, Leiden, the Netherlands) with methods as previously described.^27^ QFR ≤ 0.80 was considered significant.^34,35^

### Statistical analysis

Continuous data are expressed as mean ± standard deviation for normally distributed variables or as median [quartile (Q)1–Q3] for non-normally distributed variables. Normality was assessed using the Shapiro–Wilk test. Categorical data are presented as counts and percentages (%). Paired *t*-tests were used to compare the myocardial oxygenation response of HVBH (B-MORE_max_) with that of adenosine (A-MORE) at the patient level, with Cohen’s dz used to estimate the effect size. To account for the within-patient correlation of segmental OS-CMR data, linear mixed-effects models were then used to assess segmental OS-CMR responses. Analyses were stratified by CAD status at the global and within-territory level, and an interaction term between method and CAD presence was included to test for effect modification at the segmental level. Intraclass correlation coefficients (ICC) with 95% confidence intervals (CI) were calculated using a two-way mixed model for absolute agreement based on single measures to assess segmental SI. Inter-observer reliability was evaluated by a second observer (>15 years’ CMR experience) in a random subset of 11 paired scans at baseline and peak stress (total 264 segments). Intra-observer reliability was assessed by re-analysis of the same subset by the primary reader, blinded to previous results, and following an interval of 12 weeks. A *P*-value <0.05 was considered statistically significant. Statistical analysis was performed using the Statistical Package for Social Sciences version 29.0 (IBM Corp., Armonk, NY, USA).

## Results

### Subject characteristics

**Figure 1:**
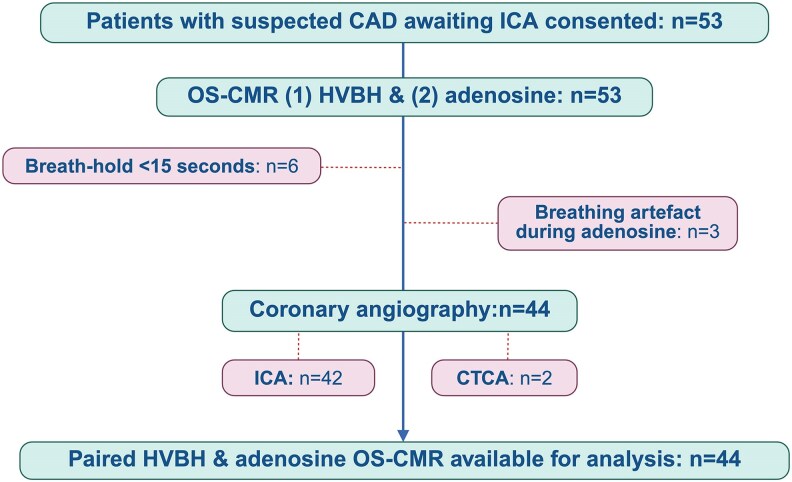
Study flow diagram. Abbreviations: CAD, coronary artery disease; CTCA, computed tomography coronary angiography; ICA, invasive coronary angiography; HVBH, hyperventilation breath-hold; OS-CMR, oxygenation-sensitive cardiovascular magnetic resonance.

Forty-four patients with complete, paired HVBH and adenosine OS-CMR sequences were included in the final analysis (mean age 69 ± 9 years, 75% male) (*Figure 1*). Nine patients were excluded: six had an insufficient voluntary breath-hold and had only the post-HV ES phase available for analysis, and three were excluded due to severe breathing artefact during adenosine stress. Baseline characteristics are summarized in *Table 1*. Participants had a high prevalence of cardiovascular risk factors and prescription medication use. CMR volumetric analysis demonstrated normal mean left ventricular volumes and systolic function. On qualitative LGE assessment, infarction was evident in 12% of patients and non-ischaemic focal fibrosis in 12%. From angiographic analysis, significant CAD prevalence was 59%. The median interval between CMR and ICA was 45 [27–72] days.

**Table 1:** Baseline characteristics

Participants with suspected angina undergoing ICA, *n* = 44	
Demographics	
Age, years	69 ± 9
Male	33 (75%)
Body mass index, kg/m^2^	29.9 ± 8.3
Cardiovascular risk factors	
Current or ex-smoker	26 (59%)
Hypertension	26 (59%)
Type II diabetes	9 (20%)
Hypercholesterolemia	13 (30%)
Family history of premature CAD	15 (34%)
Previous myocardial infarction	3 (7%)
Medications	
Aspirin	36 (82%)
P2Y12 inhibitor	3 (7%)
Statin	40 (91%)
ACEi/ARB	19 (43%)
Beta blocker	29 (66%)
Calcium channel blocker	19 (43%)
Nitrate	31 (70%)
Left ventricular function	
Ejection fraction, %	59.1 ± 7.3
End-diastolic volume index, mL/m^2^	81.4 ± 14.2
End-systolic volume index, mL/m^2^	33.4 ± 9.2
Mass index, g/m^2^	46.4 ± 8.9
LGE^a^	
Infarction	5 (12%)
Total infarcted segments (32-segment score)	22 (1.6%)
Enhanced mass, g^b^	6.2 [1.7–11.3]
Non-ischaemic focal fibrosis	5 (12%)
Insertion point fibrosis only	11 (26%)
Coronary angiography^c^	
Functionally significant stenosis	26 (59%)
Single-vessel disease	13 (30%)
Double-vessel disease	6 (14%)
Triple-vessel disease	7 (16%)
Complete or subtotal occlusion	7 (16%)

Data presented as mean ± SD, median [Q1–Q3] or absolute value (%).

Abbreviations: ACEi/ARB, angiotensin-converting enzyme inhibitor/angiotensin receptor blocker; CAD, coronary artery disease; ICA, invasive coronary angiography; LGE, late gadolinium enhancement.

^a^LGE not available: *n* = 1.

^b^In those with infarction.

^c^Invasive coronary angiography not available in *n* = 2; however, computed tomography coronary angiography performed within 1 week of CMR, which demonstrated non-obstructive CAD in both patients (diameter stenosis <50% in all epicardial vessels ≥2 mm diameter).

### Baseline haemodynamics, response to adenosine infusion and breath-hold duration

From the baseline ECG, one patient was in atrial fibrillation, with the remainder in sinus rhythm. Supplementary data online, *Table S1* summarizes the haemodynamic response to adenosine. Forty-nine per cent of participants (*n* = 21) required an adenosine dose increase, with 23% (*n* = 10) requiring the maximum dose of 210 μg/kg/min. At peak adenosine stress, 91% (*n* = 39) of participants achieved a satisfactory heart rate response ≥10 beats per minute, with a mean increase of 18 ± 8 beats per minute. Following HVBH, mean breath-hold duration was 53 ± 23 s [95% CI: 46, 60], with a mean peak oxygenation response (B-MORE_max_) at 39 ± 20 s [95% CI: 33, 45].

### OS-CMR

#### Image quality

For adenosine OS-CMR, image quality was rated as ‘excellent’ in 73% (*n* = 32), ‘good’ in 11% (*n* = 5), and ‘moderate’ in 16% (*n* = 7). For HVBH OS-CMR, the breath-hold images achieved ‘excellent’ ratings in 71% (*n* = 31), ‘good’ in 27% (*n* = 12), and ‘moderate’ in 2% (*n* = 1). For adenosine OS-CMR, three segments were excluded, and for HVBH OS-CMR one segment was excluded due to the presence of artefact.

#### Baseline myocardial oxygenation and response to hyperventilation

**Figure 2:**
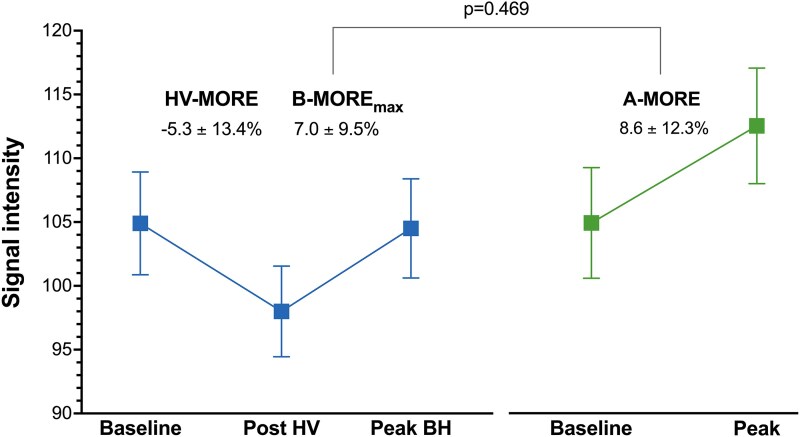
Comparison of the global myocardial oxygenation response to HVBH and adenosine. Graphical data presented as mean ± SEM. Abbreviations: A-MORE, adenosine-induced myocardial oxygenation reserve; BH, breath-hold; B-MORE_max_, breathing-induced myocardial oxygenation reserve (defined by the maximal signal intensity change during the post-hyperventilation breath-hold); HV, hyperventilation; HV-MORE, hyperventilation-induced myocardial oxygenation reserve (defined by the maximal signal intensity change following hyperventilation).

**Figure 3:**
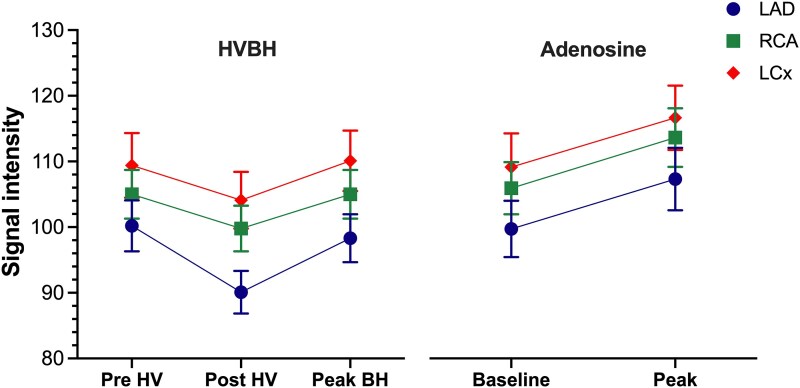
Comparison of the myocardial oxygenation response to HVBH and adenosine within individual coronary territories. Graphical data presented as mean ± SEM. Abbreviations: BH, breath-hold; HV, hyperventilation; HVBH, hyperventilation breath-hold; LAD, left anterior descending artery; LCx, left circumflex artery; RCA, right coronary artery.

Comparison of baseline OS-CMR SI prior to HVBH and adenosine administration revealed no significant differences, either globally or within individual coronary territories (see Supplementary data online, *Table S2* and *Figures 2 and 3*). Following a period of paced HV, there was a significant reduction in SI compared to baseline for the HVBH manoeuvre (see Supplementary data online, *Table S3* and *Figure 2*).

#### Myocardial oxygenation response to HVBH and adenosine

**Figure 4:**
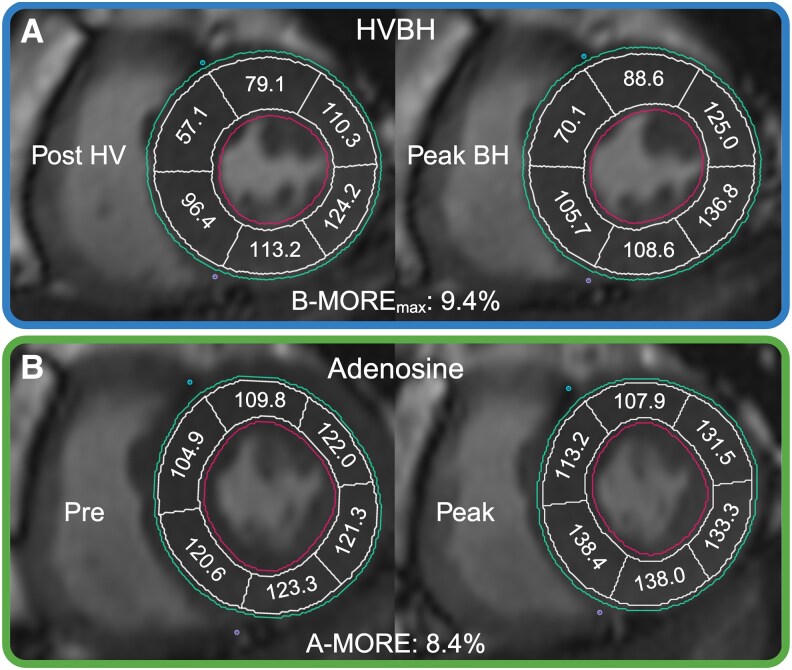
Paired comparison of the myocardial oxygenation response between HVBH (*A*) and adenosine (*B*). OS-CMR SI recorded at the segmental level, with B-MORE_max_ calculated as the maximal ΔSI% during the post-hyperventilation breath-hold and A-MORE as the ΔSI% during peak hyperaemia with adenosine. Abbreviations: A-MORE, adenosine-induced myocardial oxygenation reserve; B-MORE_max_, breathing-induced myocardial oxygenation reserve; HVBH, hyperventilation breath-hold; OS-CMR, oxygenation-sensitive cardiovascular magnetic resonance; SI, signal intensity.

Both HVBH and adenosine elicited significant myocardial oxygenation responses, which were comparable at the global level (B-MORE_max_ 7.0 ± 9.5% vs. A-MORE 8.6 ± 12.3%, mean difference: −1.6% [−6.0%, 2.8%]; *P* = 0.469) (*Table 2* and *Figures 2* and *4*). Furthermore, at the vessel level, there was a comparable myocardial oxygenation response between HVBH and adenosine within individual coronary territories (*Table 2* and *Figure 3*).

**Table 2:** Comparison of the myocardial oxygenation response to HVBH and adenosine

*n* = 44	B-MORE_max_ (%)	A-MORE (%)	Mean difference (%) [95% CI]	*P* value	Cohen’s dz	95% CI (dz)
Global	7.0 ± 9.5	8.6 ± 12.3	−1.6 [−6.0, 2.8]	0.469	−0.110	−0.406, 0.187
LAD	9.3 ± 11.9	8.8 ± 13.6	0.5 [−4.8, 5.8]		0.029	−0.267, 0.324
RCA	5.9 ± 10.1	8.1 ± 14.3	−2.2 [−7.0, 2.6]		−0.139	−0.435, 0.159
LCx	6.5 ± 10.9	9.0 ± 16.2	−2.5 [−8.1, 3.0]		−0.138	−0.434, 0.160

Data presented as mean ± SD and mean difference [95% CI].

Abbreviations: A-MORE, adenosine-induced myocardial oxygenation reserve; B-MORE_max_, breathing-induced myocardial oxygenation reserve (defined by the maximal signal intensity change during the post-hyperventilation breath-hold); LAD, left anterior descending artery; LCx, left circumflex artery; RCA, right coronary artery.

**Figure 5:**
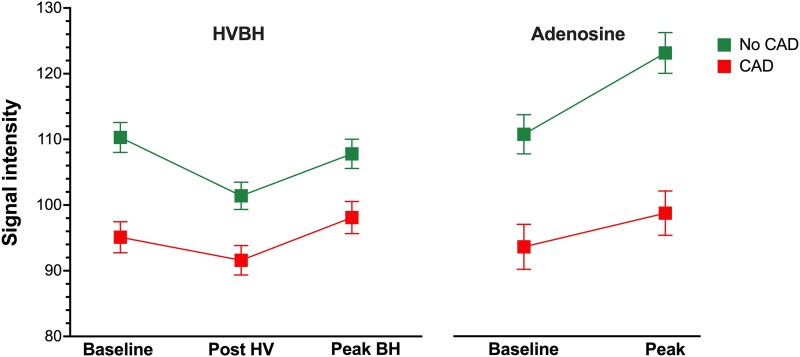
Comparison of the myocardial oxygenation response to HVBH and adenosine between territories with and without CAD. Graphical data presented as mean ± SEM. Abbreviations: BH, breath-hold; CAD, coronary artery disease; HV, hyperventilation; HVBH, hyperventilation breath-hold.

When stratifying the analysis by CAD status, patients with CAD (*n* = 26) demonstrated a B-MORE_max_ response comparable to that of A-MORE, both at the global level (7.3 ± 10.7% vs. 6.2 ± 12.4%, mean difference: −1.1% [−4.9%, 7.2%]; *P* = 0.699) and within individual coronary territories. In contrast, in patients without CAD (*n* = 18), there was a trend towards a greater A-MORE response than B-MORE_max_, though this was not statistically significant (*Table 3* and *Figure 5*).

**Table 3:** Comparison of the myocardial oxygenation response to HVBH and adenosine stratified by the presence of CAD

	B-MORE_max_ (%)	A-MORE (%)	Mean difference (%) [95% CI]	*P* value	Cohen’s dz	95% CI (dz)
CAD, *n* = 26						
Global	7.3 ± 10.7	6.2 ± 12.4	1.1 [−4.9, 7.2]	0.699	0.077	−0.309, 0.461
LAD	11.5 ± 13.0	6.3 ± 15.2	5.3 [−2.0, 12.5]		0.293	−0.102, 0.683
RCA	6.0 ± 11.6	5.0 ± 15.0	1.1 [−5.3, 7.4]		0.067	−0.318, 0.451
LCx	5.3 ± 11.5	7.2 ± 13.6	−1.9 [−8.7, 5.0]		−0.111	−0.496, 0.276
No CAD, *n* = 18						
Global	6.5 ± 7.6	12.0 ± 11.5	−5.5 [−12.0, 0.9]	0.089	−0.425	−0.903, 0.064
LAD	6.0 ± 9.4	12.4 ± 10.5	−6.4 [−13.5, 0.8]		−0.441	−0.920, 0.049
RCA	5.6 ± 7.9	12.5 ± 12.5	−6.9 [−14.4, 0.6]		−0.458	−0.939, 0.034
LCx	8.2 ± 10.0	11.6 ± 19.5	−3.4 [−13.6, 6.8]		−0.167	−0.630, 0.301

Data presented as mean ± SD and mean difference [95% CI].

Abbreviations: A-MORE, adenosine-induced myocardial oxygenation reserve; B-MORE_max_, breathing-induced myocardial oxygenation reserve (defined by the maximal signal intensity change during the post-hyperventilation breath-hold); CAD, coronary artery disease; LAD, left anterior descending artery; LCx, left circumflex artery; RCA, right coronary artery.

For segmental OS-CMR SI analysis, there was excellent agreement between observers for both HVBH (ICC 0.989 [0.984, 0.992]) and adenosine OS-CMR (ICC 0.972 [0.961, 0.980]). Similarly, there was excellent intra-observer agreement for HVBH (ICC 0.977 [0.968, 0.984]) and adenosine OS-CMR (ICC 0.969 [0.956, 0.978]).

### Segmental OS-CMR responses

In a segmental level, linear mixed-effects model adjusted for age, sex, type II diabetes, hypertension, significant CAD, and presence of segmental infarction, the difference in ΔSI% between B-MORE_max_ and A-MORE was not statistically different (ΔSI% mean difference: −1.7% [−4.0%, 0.5%]; *P* = 0.126) (*Table 4*).

**Table 4:** Adjusted associations between clinical variables and segmental myocardial oxygenation responses based on a linear mixed-effects model

Parameter	Estimate (*β*)^a^	95% CI	*P* value
Method (B-MORE_max_ vs. A-MORE)	−1.75	[−3.99, 0.49]	0.126
Age	0.14	[−0.02, 0.29]	0.077
Sex (females vs. males)	−4.92	[−8.17, −1.67]	0.003
Type II diabetes	−0.58	[−4.14, 2.98]	0.748
Hypertension	0.21	[−2.61, 3.02]	0.886
CAD	−3.59	[−6.43, −0.75]	0.013
Infarction	−0.89	[−7.94, 6.17]	0.805

Abbreviations: A-MORE, adenosine-induced myocardial oxygenation reserve; B-MORE_max_, breathing-induced myocardial oxygenation reserve; CAD, coronary artery disease.

^a^ *β* coefficients represent adjusted mean differences for categorical variables and a 1-year increase in age.

Subsequent analyses showed a significant interaction between method and CAD presence (*P* = 0.032 for interaction). Amongst patients with CAD, there was no significant difference in segmental ΔSI% between the two methods (mean difference: 1.6% [−2.2%, 5.4%], *P* = 0.400). In contrast, in those without CAD, there was a lower B-MORE_max_ response compared to A-MORE (mean difference: −3.5% [−6.3%, −0.8%]; *P* = 0.012) (*Table 5*). Additionally, within the A-MORE responses, the ΔSI% was significantly lower in those with CAD compared to those without CAD (mean difference: −6.2% [−9.9%, −2.5%]; *P* = 0.001), whereas B-MORE_max_ remained similar between those with and without CAD (mean difference: −1.0% [−4.7%, 2.6%]; *P* = 0.581) (*Table 5*). There was also no significant effect of infarction on ΔSI%.

**Table 5:** Comparison of segmental OS-CMR responses by method and CAD presence

CAD presence	Method	Adjusted mean^*^ (%)	CAD vs. No CAD (within method)	B-MORE_max_ vs. A-MORE (within CAD)
Yes	B-MORE_max_	4.5	−1.0% [−4.7%, 2.6%]; *P* = 0.581	
	A-MORE	2.9	−6.2% [−9.9%, −2.5%]; *P* = 0.001	1.6% [−2.2%, 5.4%]; *P* = 0.400
No	B-MORE_max_	5.6	Reference	
	A-MORE	9.1	Reference	−3.5% [−6.3%, −0.8%]; *P* = 0.012

^*^Adjusted for age, sex, type II diabetes, hypertension, coronary artery disease, and presence of segmental infarction. Interaction *P* = 0.032.

Abbreviations: A-MORE, adenosine-induced myocardial oxygenation reserve; B-MORE_max_, breathing-induced myocardial oxygenation reserve; CAD, coronary artery disease

## Discussion

In this prospective study, we demonstrate that in patients with suspected CAD, the HVBH manoeuvre induces changes in myocardial oxygenation comparable to those elicited by adenosine. To our knowledge, this represents the first study to evaluate these methods in patients with suspected angina. Consistent with the existing literature, our findings support the HVBH manoeuvre as an effective, alternative, non-pharmacological vasomotor stimulus, providing a potentially accessible, ‘needle-free’ approach for the assessment of myocardial ischaemia. However, this study was neither designed nor powered to determine diagnostic accuracy; therefore, the clinical utility of HVBH OS-CMR in patients with suspected CAD remains to be established.

A previous study examined the myocardial oxygenation response to vasoactive breathing manoeuvres and adenosine in 19 healthy individuals with 3-Tesla OS-CMR. The HVBH manoeuvre elicited a significantly greater response than adenosine (HVBH maximal; 14.8 ± 6.6% vs. adenosine; 3.9± 6.5%, *P* <0.05).^20^ However, limitations include the use of fixed-dose adenosine at 140 µg/kg/min, and uncertainty whether this infusion protocol resulted in a satisfactory haemodynamic response, and thus maximal hyperaemia. Furthermore, the observed adenosine ΔSI% in healthy controls without cardiovascular risk factors is considerably lower than previously reported; a previous OS-CMR study of 11 healthy controls found a ΔSI% of 21.2% [14.5%, 27.9%] following adenosine infusion.^36^

Our findings demonstrate that the HVBH manoeuvre can elicit a significant myocardial oxygenation response. This is the first study to prospectively evaluate both methods in patients with suspected CAD, a population characterized by a high burden of cardiovascular comorbidities, prescription medication use and potential coronary flow heterogeneities from obstructive CAD. Given that these factors may attenuate the myocardial oxygenation response, to ensure reliable clinical assessment of ischaemia in patients with suspected CAD, it is essential to determine whether the HVBH manoeuvre may elicit an adequate myocardial oxygenation response, comparable to that induced by the current clinical standard.

Although no prior studies have utilised OS-CMR to compare the efficacy of vasoactive breathing manoeuvres with adenosine in patients, a previous study examined the role of fast strain-encoded CMR with HVBH in 122 patients referred for clinical adenosine-stress CMR and ICA within 6 months (CAD prevalence 27%, prior revascularisation in 52%). The peak longitudinal systolic strain responses were similar between HVBH and adenosine in ischaemic segments (1.3 ± 3.8% vs. 0.6 ± 5.4%, respectively; *P* = 0.30), but differed significantly in non-ischaemic segments (−0.3 ± 3.2% vs. −0.9 ± 2.7%; *P* = 0.006). These findings are consistent with our results, with B-MORE_max_ being comparable to A-MORE in segments with CAD, while in segments without CAD, A-MORE generated a higher myocardial oxygenation response. This may be in part reflective of the distinct mechanisms by which the two methods induce coronary vasodilation. The authors further demonstrated that HVBH-strain had similar diagnostic performance to that of adenosine-strain CMR for detecting obstructive CAD, a finding which remained consistent after excluding those with prior revascularisation or infarction (*n* = 46).

Although the diagnostic performance of adenosine OS-CMR has been well established in patients with CAD,^12–16,37^ the use of the HVBH manoeuvre has thus far been limited to feasibility studies.^23,38^ In a study of 26 patients with at least one significant epicardial stenosis (≥50%), OS-CMR with HVBH revealed a blunted global ΔSI% in CAD patients compared with 10 healthy controls (2.1 ± 4.4% vs. 11.3 ± 6.1%; *P* < 0.001). Within CAD patients, there was a blunted oxygenation response in myocardial segments subtended by stenotic arteries compared with segments remote to ischaemia: 0.5 ± 3.8% vs. 3.8 ± 5.3%; *P* = 0.011.^23^ An extension of this work in 25 patients (17 of which were included in the previous study) demonstrated that patients with CAD showed an attenuated global oxygenation response at 30 s compared with controls (2.7 ± 4.8% vs. 9.1 ± 5.3%; *P* < 0.001). Moreover, in those with CAD, there were regional heterogeneities when comparing stenotic to remote territories (1.6 ± 3.9% vs. 4.9 ± 5.7%; *P* = 0.004).^38^ However, both these studies lack comparison with age-, sex-, and comorbidity-matched controls and did not include contrast-enhanced scar imaging to detect prior infarction, limiting their ability to determine whether this had an impact on the HVBH manoeuvre and myocardial oxygenation.

OS-CMR uniquely offers a non-invasive means of directly assessing the supply–demand mismatch that underpins the pathophysiology of ischaemia, making it an inherently valuable tool for evaluating patients with suspected CAD. In myocardial territories subtended by significantly stenosed arteries, the capillary bed is vasodilated at rest to maintain adequate myocardial blood flow. However, during stress, as myocardial oxygen extraction increases, there is limited capacity to further augment coronary blood flow to meet rising demand, resulting in an increased tissue deoxyhaemoglobin fraction and attenuated OS-CMR SI response.

In our study, we did not observe a significant difference in myocardial oxygenation between CAD and non-CAD patients utilising B-MORE_max_, in contrast to A-MORE, where a significant between-group difference was observed. Several factors may account for this finding. Firstly, the mechanism by which each method induces coronary vasodilation is distinct. Adenosine has an endothelial-independent action, through direct activation of A_2_A receptors resulting in smooth muscle relaxation and coronary vasodilation, leading to an uncoupling of blood flow from oxygen demand. Conversely, the HVBH manoeuvre is endothelial-dependent, relying on the rebound rise in carbon dioxide during the breath-hold phase following HV, inducing nitric oxide release and subsequent coronary vasodilation.^17,25^ However, in patients with suspected CAD, the high prevalence of coexisting cardiovascular comorbidities or concomitant microvascular dysfunction may also contribute to endothelial dysfunction, resulting in an attenuated vasodilatory response, regardless of the presence or absence of significant epicardial stenoses. Secondly, we assessed B-MORE from a single ES phase with the maximal ΔSI% response achieved during the entire voluntary breath-hold (rather than a fixed time point). Pilot work has demonstrated that fully automated analysis pipelines, which can extract multiple biomarkers over a greater number of phases and detect subtle areas of regional heterogeneity, have shown promising performance in patients with suspected CAD and angina with non-obstructive CAD. This automated workflow may provide a more robust and comprehensive understanding of the complex mechanics that underlie myocardial oxygenation.^26,39,40^

## Study limitations

Despite the prospective, comparative nature of this study, there are several limitations. Firstly, the sequence of performing the HVBH manoeuvre and administering adenosine was not randomised. Nonetheless, no significant difference was observed in baseline myocardial SI prior to performing each stress method, suggesting minimal carryover effect. The HVBH technique is dependent on patient cooperation: as such, six patients were unable to complete a sufficient breath-hold following HV and were excluded from analysis. The somewhat limited level of standardisation of the HVBH manoeuvre may introduce interindividual variability, which may impact reproducibility and image quality. However, previous studies have shown that OS-CMR SI during a post-HV breath-hold reaches a plateau within 30 s,^41^ consistent with the expected physiological responses. Moreover, the repeatability of this technique has been demonstrated before.^21^ Furthermore, as most patients in this study were in sinus rhythm, our ability to assess the impact of atrial fibrillation on image quality and the myocardial oxygenation response was limited. However, we have previously shown that the hyperaemic effects of adenosine are independent of the presence of atrial fibrillation.^42^ Therefore, it is reasonable to suggest that the oxygenation response is comparable, though this warrants future prospective evaluation. Serum haematocrit was not assessed at the time of OS-CMR (as a surrogate for hydration status) and thus could not be controlled for as a potential confounder to OS-CMR SI.^43^ Moreover, we were unable to assess the impact of concomitant coronary microvascular dysfunction, which was not assessed invasively in our study, and may lead to an underestimation of FFR and could account for discordance between impaired myocardial oxygenation and angiographically normal epicardial arteries. It may also explain the lower ΔSI% observed in females, though this remains speculative due to the small sample size. Finally, while this study provides promising comparative data validating this non-pharmacological stress method in patients, due to the absence of sufficient published data, we were unable to utilise an *a priori* definition of equivalence to power our findings.

## Conclusion

The HVBH manoeuvre induces a myocardial oxygenation response comparable to that elicited by adenosine in patients with cardiovascular comorbidities and significant CAD. These findings support the feasibility of the HVBH manoeuvre as a non-pharmacological alternative to adenosine for assessing myocardial ischaemia with OS-CMR in patients with suspected CAD. Larger, prospective studies are warranted to evaluate the diagnostic utility of HVBH OS-CMR in this population.

## Supplementary Material

qyaf141_Supplementary_Data

## Data Availability

The data underlying this article will be shared on reasonable request to the corresponding author.

## Lead author biography

Dr Simran Shergill graduated from the University of Birmingham in 2015. He completed his MRCP in 2018 and entered Cardiology specialty training in 2021. He is currently undertaking a period of research towards a PhD at the University of Leicester and the NIHR Leicester Biomedical Research Centre. His research focus involves the application of perfusion imaging and novel cardiovascular magnetic resonance techniques in patients with stable coronary artery disease.
