# Supplementary material for: Vasoactive breathing manoeuvres as an effective coronary vasodilator in patients with suspected coronary artery disease: a prospective comparative oxygenation-sensitive CMR study
Source: Eur Heart J Imaging Methods Pract. 2025 Nov 13;3(4):qyaf141. doi: 10.1093/ehjimp/qyaf141 (PMC13184620; doi:10.1093/ehjimp/qyaf141)
Supplement: qyaf141_Supplementary_Data [file qyaf141_supplementary_data.docx]

**Supplementary Table 1:** Baseline haemodynamics and response to adenosine

|  | **Baseline** | **Peak stress** | **Mean difference** | **P value** |
| --- | --- | --- | --- | --- |
| HR, bpm | 59 ± 7 | 78 ± 10 | 18 ± 8 | <0.001 |
| SBP, mmHg | 140 ± 16 | 135 ± 19 | -5 ± 15 | 0.034 |
| DBP, mmHg | 76 ± 11 | 75 ± 12 | -1 ± 11 | 0.515 |

Data presented as mean ± SD

**Abbreviations:** BPM beats per minute, DBP diastolic blood pressure, HR heart rate, SBP systolic blood pressure.

Haemodynamic data not available: n=1

**Supplementary Table 2:** Comparison of baseline OS-CMR signal intensity prior to HVBH and adenosine administration

|  | **Pre HVBH** | **Pre adenosine** | **Mean difference [95% CI]** | **P value** |
| --- | --- | --- | --- | --- |
| **Global, n=44** | 104.9 ± 26.7 | 104.9 ± 28.7 | -0.01 [-3.3, 3.2] | 0.994 |
| LAD | 100.2 ± 25.7 | 99.7 ± 28.4 | 0.5 [-3.7, 4.6] |  |
| RCA | 105.0 ± 24.6 | 105.9 ± 26.3 | -0.9 [-4.2, 2.3] |  |
| LCx | 109.4 ± 32.6 | 109.1 ± 34.0 | 0.3 [-3.1, 3.8] |  |

Data presented as mean ± SD and mean difference [95% confidence interval]

**Abbreviations:** HVBH hyperventilation breath-hold, LAD left anterior descending artery, LCx left circumflex artery, OS-CMR oxygenation-sensitive cardiovascular magnetic resonance, RCA right coronary artery.

| **n=44** | **Pre HV SI** | **Post HV SI** | **Mean difference [95% CI]** | **HV-MORE** | **P value** |
| --- | --- | --- | --- | --- | --- |
| **Global** | 104.9 ± 26.7 | 98.0 ± 23.6 | 6.9 [2.6, 11.1] | -5.3 ± 13.4% | 0.002 |
| LAD | 100.2 ± 25.7 | 90.1 ± 21.5 | 10.1 [6.2, 14.0] | -8.8 ± 13.1% |  |
| RCA | 105.0 ± 24.6 | 99.8 ± 23.2 | 5.2 [0.9, 9.4] | -3.9 ± 13.6% |  |
| LCx | 109.4 ± 32.6 | 104.1 ± 28.7 | 5.4 [-0.2, 10.9] | -3.0 ± 16.2% |  |

**Supplementary Table 3:** Myocardial oxygenation response following a period of paced hyperventilation prior the voluntary breath-hold of the HVBH manoeuvre

Data presented as mean ± SD and mean difference [95% confidence interval]

**Abbreviations:** HV hyperventilation, HVBH hyperventilation breath-hold, HV-MORE hyperventilation-induced myocardial oxygenation reserve, LAD left anterior descending artery, LCx left circumflex artery, RCA right coronary artery, SI signal intensity.
